# Clinical and hormonal characteristics of patients with different types of hypophysitis: a single-center experience

**DOI:** 10.20945/2359-3997000000102

**Published:** 2019-02-01

**Authors:** Narin Nasiroglu Imga, Ali Erdem Yildirim, Ozden Ozdemir Baser, Dilek Berker

**Affiliations:** 1 Saglik Bilimleri University Ankara Numune Education and Research Hospital Department of Endocrinology and Metabolism Ankara Turkey Saglik Bilimleri University, Ankara Numune Education and Research Hospital Department of Endocrinology and Metabolism, Ankara, Turkey; 2 Saglik Bilimleri University Ankara Numune Education and Research Hospital Department of Neurosurgery Ankara Turkey Saglik Bilimleri University, Ankara Numune Education and Research Hospital Department of Neurosurgery, Ankara, Turkey

**Keywords:** Primary hypophysitis, secondary hypophysitis, hypopituitarism, diabetes insipidus

## Abstract

**Objective:**

The inflammation of the pituitary gland is known as hypophysitis. It is a rare disease accounting for approximately 0.24%-0.88% of all pituitary diseases. The natural course of hypophysitis is variable. Main forms are histologically classified as lymphocytic, granulomatous, IgG4 related and xanthomatous. We aim to present our patients with hypophysitis and compare clinical, laboratory and radiological features.

**Subjects and methods:**

We retrospectively reviewed our database of 1.293 patients diagnosed with pituitary diseases between 2010 and 2017. Twelve patients with hypophysitis were identified. Demographical data, clinical features, endocrinological dysfunction, imaging findings, treatment courses and follow-up periods were evaluated.

**Results:**

The frequency of hypophysitis was found 0.93% in all cases of the pituitary disease. Twelve patients (nine females and three males), ages between 17-61 years, were evaluated. The characteristic features of our patients tended to be predominantly female and young. Diagnosis of hypophysitis was made after pituitary biopsy in four patients, and in eight patients after pituitary operation due to adenoma. Headache (67%) and visual problems (33%) were the most frequent nonendocrine symptoms. Anterior pituitary hormone deficiencies (63.7%) and/or diabetes insipidus (17%) were seen among patients. According to histopathological forms, four had lymphocytic, seven had granulomatous and one had xanthogranulomatous types. Contrast enhancement heterogeneous and thickened pituitary stalk were the most common radiological alterations.

**Conclusion:**

Hypophysitis should be considered in the differential diagnosis of sellar masses. It can mimic pituitary adenomas in radiological and endocrinological aspects. The different patterns of pituitary hormone deficiencies may be seen in the course of the disease.

## INTRODUCTION

Focal or diffuse inflammation and cellular infiltration of the pituitary gland is known as hypophysitis. It is a significant condition, which can mimic the other sellar lesions and result in the destruction of pituitary gland cells. It may also result in isolated anterior and posterior pituitary hormone deficiency or panhypopituitarism (1). Hypophysitis is an uncommon disease accounting for approximately 0.24%-0.88% of all pituitary diseases, with a reported annual incidence rate of one case per 9 million (2–4). Based on histological appearance and pathophysiological mechanism, the inflammation of the pituitary gland is categorized as primary and secondary hypophysitis. Secondary hypophysitis can be originated from neighboring tissue lesions or multisystemic inflammatory diseases (5,6). Primary hypophysitis is classified as lymphocytic (autoimmune), granulomatous, xanthomatous, IgG4-related and lymphoplasmacytic. It also has mixed forms called lymphogranulomatous and xanthogranulomatous hypophysitis. Xanthomatous hypophysitis is characterized by infiltration of foamy histiocytes in the anterior pituitary gland. Causes and natural courses of secondary hypophysitis are variable. According to the anatomical location, hypophysitis lesions are classified into adenohypophysitis, infundibuloneurohypophysitis or panhypophysitis (7).

In this study, we aim to present clinical features, neurologic and endocrinologic data, and treatment courses of twelve patients with hypophysitis that followed up in a single-center.

## SUBJECTS AND METHODS

We retrospectively reviewed our database of 1.293 patients diagnosed with a pituitary mass disease followed at Ankara Numune Education and Research Hospital Department of Endocrinology between 2010 and 2017. Out of 1.293 patients, 12 patients who had histological confirmation of hypophysitis were included to the study. Demographical and laboratory data, clinical features, endocrinological dysfunction, magnetic resonance imaging (MRI) findings of the pituitary gland, treatment courses and follow-up period were all evaluated. All patients had initial and control MRI images taken of their pituitary gland. Pituitary gland volume was measured by the volume = 1/2 x length x width x height formula, as described in a previous study (8). All patients underwent transsphenoidal microscopic surgery (TSS). After TSS, all specimens were exposed to hematoxylin and eosin (H&E) staining and immunohistochemical staining. After that, according to underlying disease, they were divided into primary or secondary hypophysitis. By the anatomical location, the cases were classified into adenohypophysitis, infundibuloneurohypophysitis or panhypophysitis. Pretreatment and post-treatment plasma concentrations of hormones related to the pituitary gland, such as ACTH, basal cortisol, thyrotropin stimulating hormone (TSH), free T4, free T3, growth hormone (GH), IGF-1, prolactin, follicle-stimulating hormone (FSH), luteinizing hormone (LH) and estradiol or total and free testosterone levels, were evaluated. In addition, the function of the posterior pituitary gland was evaluated with clinical symptoms according to plasma and urinary osmolality and plasma sodium levels. The laboratory analyses of all these cases were evaluated. Growth hormone deficiency (GHD) was diagnosed by the insulin tolerance test (ITT) or glucagon test. In patients with clear-cut characteristics of GHD and three other documented pituitary hormone deficits the diagnosis of GHD was made without biochemical testing. Steroid replacement therapy was given to patients with adrenal insufficiency. Adrenal insufficiency diagnosed if a morning cortisol level < 3 μg/dL and a level > 15 μg/dL likely excluded an adrenal insufficiency diagnosis. In case of cortisol levels between 3-15 μg/dL adrenal insufficiency was assessed using ITT or standard-dose ACTH stimulation tests. Peak cortisol levels > 18.1 μ/dL at 30 or 60 minutes excluded adrenal insufficiency. Central hypothyroidism diagnosis confirmed by free T4 level below the laboratory reference range in concurrence with a low, normal, or mildly elevated TSH (9). For the follow-up in the first year, the patients were evaluated every six months after the surgery, followed by every year. The local ethical committee of Ankara Numune Education and Research Hospital approved the study.

## RESULTS

The frequency of hypophysitis was found 0.9% among our 1.293 cases with pituitary disease. The follow-up time ranged from 6 to 98 months (median: 28 months). Demographical characteristics of 12 cases and pituitary gland volume at the presentation and control are summarized in Table 1. Twelve patients (nine females and three males) with ages ranged between 17 and 61 years (mean age: 44) were evaluated. At the time of diagnosis female patients were not pregnant or in the puerperium period. The mean age at presentation was between fourth and fifth decades in lymphocytic and granulomatous hypophysitis; on the other hand, a case of xanthogranulomatous hypophysitis was younger (27 years). The features suggesting hypophysitis on the MRI scans were: diffuse symmetric enlargement of the pituitary gland, a thickened stalk, expansion of sella turcica and homogeneous or heterogeneous pituitary gland. Mean pituitary gland volume at presentation was found to be 6,115 ± 8,873 mm^3^. However, in the follow-up period, the mean pituitary gland volume was decreased to 1,009 ± 2,225 mm^3^. No other major changes were observed radiological image pattern. Pituitary surgery was performed for histological diagnosis and to improve the compressive neuropathy symptoms in big-sized pituitary gland and mimicking adenoma cases. In cases of a small-sized pituitary gland, surgery was performed only for histological diagnosis. In four patients diagnosis was made through pituitary biopsy, while in eight patients after pituitary operation. All the patients underwent TSS, and histopathological diagnosis was made through H&E staining and immunohistochemical staining. According to the histopathological features, three types of the hypophysitis were detected: lymphocytic (n = 4), granulomatous (n = 7) and xanthogranulomatous (n = 1). The clinical characteristics, radiological and endocrinologic findings of patients according to histological diagnosis of hypophysitis was given in Table 2.

**Table 1:** Demographical data of patients according to those subgrouped with histological diagnosis

		All	LyHy (n = 4)	GrHy (n = 7)	XantgrHy (n = 1)
Mean age, y		44 (17-61)	40 (17-55)	48 (28-61)	27
Gender					
	Female	9 (75%)	3 (75%)	6 (%86)	–
	Male	3 (25%)	1 (%25)	1 (%14)	1 (100%)
BMI		26.8 ± 2.6	26.2 ± 2.4	26.9 ± 2.9	29.1
Pituitary gland volume baseline (mm^3^)		6,115 ± 8,873	8,169 ± 7,717	5,564 ± 10,403	1,755
Pituitary gland volume after follow-up (mm^3^)		1,009 ± 2,225	4,415 ± 3,867	174 ± 315	40.5

LyHy: lymphocytic hypophysitis; GrHy: granulomatous hypophysitis; XantgrHy: xanthogranulomatous hypophysitis BMI: body mass index.

Values are expressed as mean (range), median or number (%).

**Table 2:** The clinical characteristics, radiological and endocrinologic findings of patients according to histological diagnosis of hypophysitis

Symptoms and clinical signs	All (n = 12)	LyHy (n = 4)	GrHy (n = 7)	XantgrHy (n = 1)
Headache	8 (67%)	2 (50%)	5 (71%)	1 (100%)
Weight gain	2 (16%)	1 (25%)	1 (14%)	0
Nausea, vomiting	3 (25%)	1 (25%)	2 (29%)	0
Visual defects	4 (33%)	2 (50%)	2 (29%)	0
Amenorrhea/impotence	5 (41%)	1 (25%)	3 (43%)	1 (100%)
Initial radiological signs				
Pituitary gland swelling	4 (33%)	2 (50%)	2 (29%)	–
Compression of the optic chiasm	4 (33%)	1 (25%)	3 (43%)	–
Thickened pituitary stalk	5 (41%)	2 (50%)	3 (43%)	–
Contrast enhancement heterogeneous	7 (58%)	2 (50%)	4 (57%)	1 (100%)
**Initial endocrinological disturbance**				
Diabetes insipidus	2 (17%)	1 (25%)	1 (14%)	–
Hypogonadism	9 (75%)	3 (75%)	6 (86%)	–
GH deficiency	6 (50%)	2 (50%)	4 (57%)	–
Hypothyroidism	9 (75%)	4 (100%)	5 (71%)	–
Adrenal insufficiency	7 (58%)		4 (57%)	–
Hyperprolactinemia	1 (8%)	1 (25%)	1 (14%)	–

LyHy: lymphocytic hypophysitis; GrHy: granulomatous hypophysitis; XantgrHy: xanthogranulomatous hypophysitis; GH: growth hormone.

Symptoms were found non-specific, but the most predominant non-endocrine symptoms were headache (67%) and visual field defects (33%), followed by secondary amenorrhea and impotence. The duration of symptoms varied from a few days to over five years. Pituitary gland swelling (33%), compression of the optic chiasm (33%) thickened pituitary stalk (41%) and heterogeneous contrast enhancement (58%) were identified in patients. Endocrinological evaluation was performed in all cases. Pituitary gland dysfunction was seen in variable degrees. Panhypopituitarism was seen in 50% of the cases and 25% had one or more isolated hormone deficiencies. The most isolated pituitary dysfunctions were suppressed gonadal axis, followed by suppression of pituitary-thyroid axis (75%). Diabetes insipidus (DI) was identified in 17% of patients. At the time of the diagnosis, seven patients received steroid therapy (58%) and nine patients received levothyroxine therapy (75%). Four cases with primary hypophysitis were treated with high doses of glucocorticoids (1g/day methylprednisolone for three days), followed by tapering gradually (10 mg/day). Complete recovery was observed in one patient (25%). During the follow-up period, six patients remained to receive steroid therapy, seven on levothyroxine therapy and nine on hypogonadotropic hypogonadism therapy. Radiological features of all cases at presentation were evaluated according to MRI focused on the sellar area (Table 3). Heterogeneous contrast enhancement and increased infundibulum thickening were found in 50% of lymphocytic cases and in 57% of granulomatous hypophysitis cases. Cavernous sinus invasion and optic tract compression were found in one case of lymphocytic and one case of granulomatous hypophysitis. Etiology of the hypophysitis including autoimmune diseases and antibodies were investigated in all patients. All the lymphocytic cases were found idiopathic. Granulomatous hypophysitis cases were evaluated; three patients had secondary granulomatous hypophysitis and four patients were idiopathic. All three cases of secondary hypophysitis were diagnosed with tuberculosis hypophysitis and the diagnosis of pituitary tuberculosis was made through a positive tuberculin skin test and response of an interferon-gamma release by QuantiFERON TB- Gold test. These patients received antituberculous therapy. In the follow-up period, no recurrence of hypophysitis was observed in patients. The visual functions were improved in patients who had optic tract compression. Contrarily, the hormonal deficiencies were not improved and were aggravated after the diagnosis in some patients.

**Table 3:** The clinicopathological characteristics and radiological features of the patients included in this study

Case nº	Diagnosis	Age, Gender	Size (mm)	Gland volume (cm^3^)	Enhancement	Infundibulum Thickening	Cavernous sinus invasion	Optic tract compression
1	Lymphocytic	31, F	29	4,872	Homo	Yes	No	No
2	Lymphocytic	55, F	36	19,656	Homo	Yes	Yes	Yes
3	Lymphocytic	17, F	18	5,148	Hetero	No	No	No
4	Lymphocytic	40, M	15	3,000	Hetero	No	No	No
5	Granulomatous	39, F	20	1,200	Homo	No	No	No
6	Granulomatous	37, F	28	28,910	Homo	Yes	Yes	Yes
7	Granulomatous	61, F	14	1,568	Homo	Yes	No	No
8	Granulomatous	48, F	11	495	Hetero	No	No	No
9	Granulomatous	48, F	15	750	Hetero	No	No	No
10	Granulomatous	51, F	11	1,073	Hetero	Yes	No	No
11	Granulomatous	28, M	29	4,959	Hetero	Yes	No	No
12	Xhanthogranulomatous	27, M	15	1,755	Hetero	Yes	No	No

F: female; M: male.

## DISCUSSION

The natural course of hypophysitis is variable, ranging from spontaneous recovery to death. The prevalence and incidence of hypophysitis are not known with accuracy. In previous studies, the frequency of hypophysitis tends to be more in females (10,11). Our study confirmed the male/female ratio of 1:3 with the dominance of females reported in recent studies. The hormonal and clinical manifestations of hypophysitis are variable for all types. The signs and symptoms are related to anterior or posterior pituitary hormone deficiency and compression of sella. It is estimated that the immune system affects the anterior lobe, posterior lobe or both and caused hormone deficiency (12). According to clinical and neuroradiological findings, hypophysitis can involve the anterior pituitary gland, infundibulum, posterior lobe (infundibuloneurohypophysitis) or the entire pituitary gland (panhypophysitis) (13). Infundibuloneurohypophysitis is related to the etiological diagnosis of anterior hypopituitarism, particularly GH deficit, and secondary hypogonadism and DI (14).

In other studies, it was reported that primary hypophysitis was usually seen in the anterior lobe of hypophysitis (15), and symptoms due to anterior pituitary hormone deficiency were the highest (16). This anterior pituitary deficiency was most common in our study group. Most of our patients had adenohypophysitis involvement, and DI was seen in 16% of the patients. These patients had panhypophysitis and infundibuloneurohypophysitis. In the literature, it was reported that infundibuloneurohypophysitis is a cause of central DI (17,18).

Primary hypophysitis is commonly idiopathic, but in some cases it is related to other autoimmune diseases, such as autoimmune polyglandular syndrome (APS), Hashimoto thyroiditis, Graves’ disease, type 1 diabetes mellitus, systemic lupus erythematosus and Sjögren's syndrome. Secondary hypophysitis causes are variable and consist of local pituitary lesions (adenomas, craniopharyngiomas, Rathke's cleft cysts, germinomas and meningiomas), systemic inflammatory diseases (sarcoidosis, granulomatosis with polyangiitis, vasculitis), infectious diseases (tuberculosis, syphilis, aspergillosis, coccidioidomycosis), infiltrative diseases (hemochromatosis, amyloidosis, Langerhans cell histiocytosis) and secondary to treatment (anti-CTLA-4 monoclonal antibodies, interferon-alfa), but the source is frequently unknown. Lymphocytic hypophysitis is initially characterized by lymphocytic infiltration and expansion of the pituitary gland, and followed by destruction of the pituitary cells (12). Granulomatous hypophysitis is characterized by infiltration by histiocytes and giant cells. Xanthomatous hypophysitis is a very uncommon form of hypophysitis, and histologically it is characterized by infiltration of foamy histiocytes in the pituitary gland (19). Lymphocytic hypophysitis is the most common subtype of hypophysitis (7), but the granulomatous type was the most common type seen among our patients. In our patients, 67% had granulomatous hypophysitis and 33% had lymphocytic hypophysitis. Based on the histopathologic features IgG4 related hypophysitis is a rare type of hypophysitis, but IgG4 positivity was not found in our patients.

The courses of primary and secondary hypophysitis are variable and unpredictable (20). Naturally, in the pituitary gland, inflammation primarily occurs and then is followed by edematous and an enlarged gland, and then the secondary mass effects are developed. The mechanism is due to the destruction of pituicytes, and the parenchyma is replaced by fibrosis. This results in pituitary atrophy and leads to hypopituitarism. The disease course may be rapidly destructive and aggressive (21). In some cases in the literature, the partial or full recovery resolution of pituitary functions has been recognized (22,23). In our study, full recovery was seen in only one case. Partial recovery of pituitary function was seen in 17% of patients, and 50% of patients remained unchanged in pituitary functions during follow-up. Long-term follow-up is essential for possible hypopituitarism due to development of empty sella.

In the management of hypophysitis, a symptomatic treatment approach is usually needed. Symptomatic treatment can both minimize the size of the pituitary mass and replace the defective endocrine function. Observation with replacement for deficient pituitary hormones, TSS, radiotherapy, medical treatment with immunosuppressant drugs such as glucocorticoids, azathioprine, methotrexate or cyclosporine A are treatment options to reduce the mass effect. Owing to the lymphocytolytic features, the glucocorticoids are efficient for reducing the volume of the pituitary mass and the thickened stalk (24,25). Therewithal, the treatment of secondary hypophysitis must be focused for the etiology of underlying disease and simultaneous missing hormone replacement. In addition, providing the histological diagnosis surgery can achieve sellar mass decompression and resolve the symptoms of mass effect. In our patients, of all who underwent surgical treatment, three of five patients who had lymphocytic hypophysitis who received high dose glucocorticoid treatment, and three of seven patients who had granulomatous hypophysitis who had treatment of underlying disease showed significant mass reduction. Complete recovery without any hormone replacement was observed in one patient. We consider that the diffuse lymphocytic and granulomatous infiltration leads to cell destruction in the pituitary gland and resulted in irreversible pituitary hormone dysfunction. As seen in our patients, the individuals who had hypopituitarism usually did not benefit from surgical decompression and high dose glucocorticoid treatment. After the fibrous stage of hypophysitis, the responsiveness of glucocorticoid therapy is decreased.

Surgical treatment is generally appropriate for achieving decompression of pituitary masses, however, it infrequently improved hormone deficiency. It is suggested that surgery should generally be performed in the presence of critical and progressive deficits of visual fields and in patients who are unresponsive to medical treatment. Surgery also has some complications, such as bleeding, cerebrospinal fluid leaks and DI (26). Consequently, in the treatment of hypophysitis in the absence of symptoms related to mass effect, the replacement of missing hormones must be performed. After that, hypophysitis should be distinguished as primary or secondary. If the diagnosis of primary hypophysitis is made, high dose glucocorticoid therapy should be given, for the secondary hypophysitis appropriate management of underlying disease still remains incontrovertible.

Pituitary tuberculosis is a rare cause of a secondary hypophysitis. All our three cases of secondary hypophysitis were diagnosed with tuberculosis hypophysitis, without the systemic manifestations of tuberculosis infection. The histological studies showed chronic inflammatory lesions of granulomatous type in different phases, leading to different degrees of destruction and necrosis of the pituitary gland with giant cells. The diagnosis of pituitary tuberculosis was made through a positive response of an interferon-gamma release by QuantiFERON TB- Gold test and positive tuberculin skin test. A confirmation by acid fast Bacilli, and chain polymerase reaction for detection of mycobacterial DNA, and Ziehl-Neelsen staining tests of pituitary biopsy were found negative. Patients received antituberculous therapy at least one year. In the follow-up period, no recurrence of hypophysitis was observed in patients.

The limitation of our study is the lack of serum anti-pituitary auto-antibodies (APA) and serum anti-hypothalamus auto-antibodies (AHA) because these tests have not been routinely available in our laboratory. It was suggested that high positivity of APA and AHA has the potential to be helpful in the diagnosis of hypophysitis (27) but autoantigens are poorly defined (28).

In conclusion, hypophysitis should be kept in mind in patients with hormone deficiencies and sellar masses. It can mimic pituitary adenomas in radiological and endocrinological aspects. The different patterns of anterior pituitary hormone deficiencies and diabetes insipidus may be seen in the course of disease. The deficit of the anterior pituitary hormones was seen most commonly in our cases of hypophysitis. In the absence of surgical decompression, the first-line therapy must be the replacement of missing hormones. Surgical treatment should be done to reduce the size of the mass and degree of compressive neuropathy, such as ophthalmoplegia and visual field defects. The goal of the medical treatment is consistent with high dose glucocorticoids for primary hypophysitis, and for secondary hypophysitis is underlying disease. Due to possible development of empty sella long-term, follow-up is necessary in all hypophysitis cases.
